# High Temperature Sensing and Detection for Cementitious Materials Using Manganese Violet Pigment

**DOI:** 10.3390/ma13040993

**Published:** 2020-02-22

**Authors:** Rajagopalan Sam Rajadurai, Jong-Han Lee

**Affiliations:** Department of Civil Engineering, Inha University, Incheon 22212, Korea; samraj9593@gmail.com

**Keywords:** irreversible thermochromic, cement composite, manganese violet, temperature indication, heat monitoring

## Abstract

In recent years, advanced materials have attracted considerable interest in the field of temperature detection and sensing. This study examined the thermochromic properties of inorganic manganese violet (MV) with increasing temperature. According to the thermochromic test, the material was found to have reversible and irreversible color change properties. The MV pigment was then applied to cementitious material at ratios of 1%, 3%, and 5%. The mixed cement samples with MV pigment were heated in a furnace, and digital images were captured at each temperature interval to evaluate the changes in the color information on the surface of the specimen. The mixed samples exhibited an irreversible thermochromic change from dark violet to grayish green above 400 °C. At the critical temperature of 440 °C, the *RGB* values increased by approximately 22%–55%, 28%–68%, and 7%–25%, depending on the content of MV pigment. In Lab space, the *L* value increased by approximately 23%–60% at 440 °C. The *a* value completely changed from positive to negative, and the *b* value changed from negative to positive. All the values differed according to the content of MV pigment at room temperature but approached similar ranges at the critical temperature, irrespective of the amount of MV pigment. To assess the changes in their microstructure and composition, scanning electron microscopy and energy dispersive X-ray spectroscopy were performed on the samples exposed to temperatures ranging from room temperature to 450 °C.

## 1. Introduction

Cementitious materials are some of the most essential and reliable construction materials used in a range of applications, from building, bridges, and underground structures to special structures, such as nuclear power plants, chimneys, and reactors. The cement-based structures are normally exposed to room temperature but can sometimes be exposed to high temperature environments. When cementitious materials and structures are exposed to high temperatures, the material and mechanical properties can deteriorate, such as decreases in compressive and tensile strength, elastic modulus, cracking, and spalling [1,2]. In addition, complex physical and chemical changes occur when exposed to elevated temperatures [3,4]. Primarily, the physically combined water begins to evaporate, and the C-S-H (Calcium-Silicate-Hydrate) starts to dehydrate at approximately 100 °C [5,6]. The C-S-H decomposes gradually between around 100 °C to 300 °C [6]. From approximately 500 °C, the portlandite begins to disintegrate, and the calcite begins to decompose [3,6]. These phenomena lead to a deterioration of the material, such as a decay of strength and elastic modulus, as well as cracking and spalling of concrete.

After the cement-based structures are damaged by high temperatures, most of the structures can work perfectly after proper repair and maintenance instead of rebuilding the structures. To ensure that a structure can be repaired or replaced, an appropriate evaluation method is needed to define the damaged part of the structure due to the high temperature. At the same time, the evaluation should be fast and easy after an event. Typically, core strength and rebound hammer tests are performed to define the strength decay of the high-temperature damaged structures. Furthermore, the ultrasonic pulse velocity and the impact echo method can be used to detect the degree of damage. Similarly, some techniques, such as infrared thermal imaging, drilling resistance, petrographic examination, thermoluminescence, and the Windsor probe test, also detect damage to concrete [7,8,9]. On the other hand, these non-destructive techniques provide an indirect indication of damage to the structure and have limited application to the wide and large area of a real structure in practice.

The evaluation of high-temperature damaged structures also uses color changes. Normal cementitious materials containing siliceous aggregate tend to change color to pink or red at 300–600 °C, to whitish gray at 600–900 °C, and to buff at 900–1000 °C [10,11,12,13,14]. Moreover, the strength of concrete begins to decrease from 300 °C and drops by approximately 50% to 60% at approximately 500 °C [15,16]. Therefore, a color change to pink or red can indicate a deterioration of the material and mechanical properties. However, the degree of color change is difficult to define and recognize, particularly by the naked eye. Moreover, the color changes are less prominent for calcareous and igneous aggregates. Therefore, some studies based on color changes roughly estimated the temperature of the cementitious materials and were lack in the application to define the temperature information.

Color is the most immediate visible aspect in terms of exposure to high temperatures. To define the temperature information more precisely, a color change pigment was essentially mixed with cementitious materials. Some materials have a property to change color in response to the exterior environment, such as temperature and light [17]. The materials, which were recently recognized to exhibit a distinct color change in response to change in temperature, are found in different forms, such as metal oxides, polymers, pigments, and leuco dyes. Reversible thermochromic materials are used in the applications of smart windows, ultra-thin films, temperature sensors, cool colored, and protective coatings [18,19,20,21,22,23,24,25,26]. The irreversible thermochromic materials experience a permanent color change at certain temperatures and are widely used in applications, such as aeronautical components, furnaces, damage warnings, and thermal flow sensors [27,28,29]. Previous studies focused on metal structures but not on cementitious materials and structures widely used in the construction field.

In this study, manganese violet pigment was identified in the furnace test to undergo an efficient color change from dark violet to grayish yellow with temperatures above 400 °C. The manganese violet pigment, which is composed of manganese dioxide, ammonium dihydrogen phosphate, and phosphoric acid, presents a dark violet color due to the presence of phosphate and ammonia [30,31]. The color change to grayish yellow results from the evaporation of water and the liberation of ammonia from the pigment particles. The manganese violet pigment was then applied to white cement at a ratio of 1%, 3%, and 5% of the total mass. The white cement is similar to ordinary Portland cement except for the color. The white color was attributed to the decrease in the amount of chromium, manganese, iron, copper, vanadium, nickel, and titanium in the ordinary Portland cement. The white cement confers an advantage for the excellent exposure of color when mixed, and the material properties are unaffected due to a decrease in the above components [32]. The pigment mixed samples were heated in a furnace from room temperature to 450 °C. The mixed samples underwent a typical thermochromic change to a grayish green color. The thermochromic change was observed visually by the naked eye and was evaluated using digital images captured at each temperature interval.

Digital images are typically recorded in *RGB* color space, which is widely used in cameras and monitors by the percentage of red, green, and blue constituents. The *RGB* values of each component are in the range of zero to 255 [33]. The disadvantage of *RGB* color space is dependent on each device, which has its own different color sensor characteristics and produces different *RGB* output responses. Unlike the *RGB*, *Lab* color space can be used as a device-independent model when representing color. The color information in both *RGB* and *Lab* color spaces obtained from the digital images were analyzed, and the color information with temperatures was quantified. From the above techniques, a reliable method for temperature detection can be obtained by integrating the color change data and with those of the microstructural and the compositional changes. The microstructure changes caused by the high temperatures were studied by scanning electron microscopy (SEM). In addition, the changes in their composition due to the high temperature were examined using energy dispersive X-ray spectroscopy (EDX). Therefore, this study analyzed the color information visually, the changes in the *RGB* and *Lab* color spaces obtained from the digital images, and the microstructure and compositional changes with the application of high temperatures. 

## 2. Experimental Program

### 2.1. Specimen Preparation and Test Procedure

For the application of manganese violet (MV) pigment [34] to the sensing and detection of high temperature in cementitious materials, this study conducted the thermochromic tests of MV pigment and mixed samples. Table 1 lists the typical characteristics of MV pigment. The MV pigment sample was first prepared and placed in a porcelain crucible for the thermochromic test. The pure sample was heated from room temperature to 450 °C in an electrical furnace. The color of the sample was examined at 100 °C, 200 °C, 300 °C, and 350 °C. Due to the fact that the sample was expected to change its color above 350 °C, the change in the color was examined from 350 °C to 450 °C at 10 °C intervals. The pigment sample was maintained at each target temperature for approximately 10 minutes to induce a uniform temperature distribution in the sample. Subsequently, the color change on the surface of the pigment was recorded using a digital camera. In addition, cementitious mixed samples were manufactured with white cement and MV pigment. White cement is typically similar to ordinary Portland cement, but has a high degree of whiteness due to a decrease in the amount of chromium, manganese, iron, copper, vanadium, nickel, and titanium. White cement also has distinct color recognition and decreases the strength reduction when mixed with color pigments. Therefore, white Portland cement is commonly used for colored concrete and mortar combined with pigments and precast concrete members required for high early strength. 

The ACI-212 (American concrete institute) [35] requires that the addition of pigment should not exceed 10% of the weight of the cementitious material and recommends less than 6% to have little or no effect on the mechanical properties of fresh and hardened concrete, which would also be economical. Therefore, this study defined the mixed ratios of the MV pigment as 1%, 3%, and 5% of the weight of white cement. The pigment was added and thoroughly mixed under a dry condition. After mixing with cement material, thermochromic tests were carried out in an electrical furnace to examine the color change of the mixed samples with increasing temperature from room temperature to 450 °C.

After the thermochromic heating tests were performed on the MV pigment and the mixed samples, the samples were collected before and after heating to the target temperatures. The fragments of the collected samples were positioned on the sample holder to perform different investigations. SEM examined the microstructural changes, and EDX was performed to analyze the compositional changes of the samples exposed to high temperatures.

### 2.2. Color Measurement and Analysis

To investigate the thermal color alteration of the samples, a SONY ILCE-TR digital camera was installed parallel to the samples at a distance of approximately 30 cm, and pictures of the surfaces of the heated samples were taken at each temperature interval. The images taken in the normal background and stored in JPEG format were processed to quantify the color information in both *RGB* and Lab color spaces. 

The *RGB* color space is widely used for image processing and graphics resulting from cameras and monitors. In the *RGB* color space, every color is represented by the spectral components of *R* (red), *G* (green), and *B* (blue). However, the color intensities in the *RGB* space depend strongly on device sensors and characteristics, which could produce different *RGB* color output responses. On the other hand, *Lab* color space shows a representative color independent of the devices and includes all the colors in the spectrum, as well as colors outside human perception. Therefore, the *Lab* color space with high accuracy and portability is largely used in practical applications, such as printing, automobile, textiles, and plastics. In the Lab color space, the *L* value is a measure of lightness ranging from 0 to 100, which represents complete darkness or opaqueness to complete transparence, respectively. *a* and *b* range from −128 to 128. Positive and negative *a* indicate the redness and greenness, respectively, and positive and negative *b* is *a* measure of yellowness and blueness, respectively.

## 3. Thermochromic Characteristics of MV Pigment

### 3.1. Thermochromic Color Change of MV Pigment with High Temperatures

**Figure 1:**
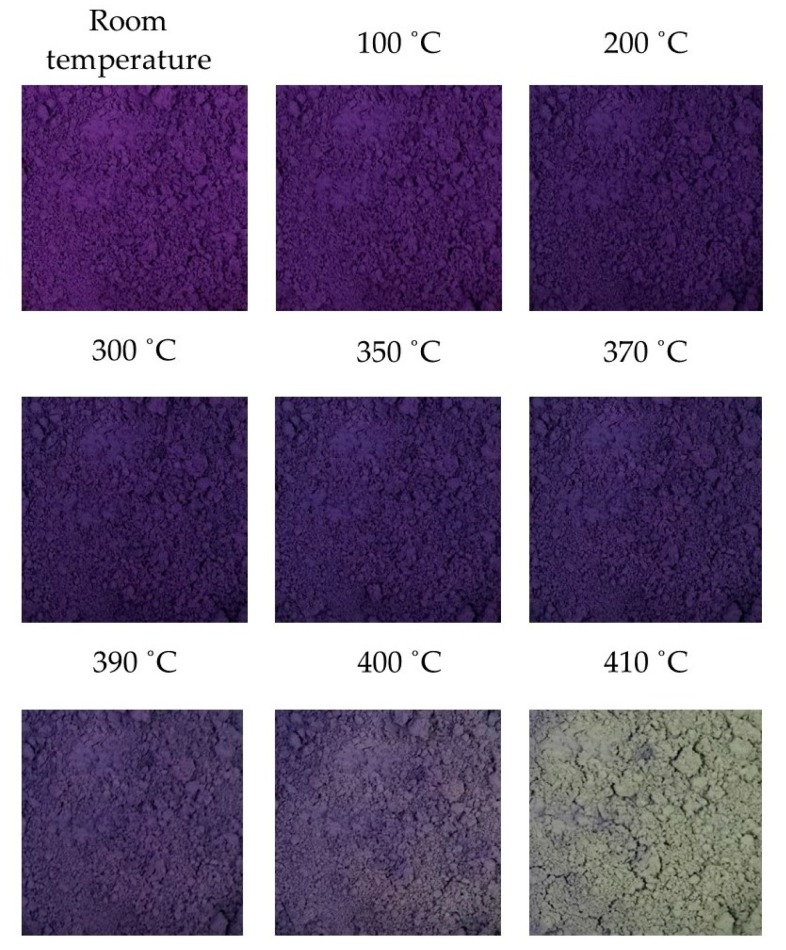
Color changes in the MV pigment with temperatures (Scale: 1000 × 1000 pixels).

As the MV pigment samples were exposed to different high temperatures, the color changes were observed with increasing temperature from room temperature. Figure 1 presents the color changes of the samples at each temperature. The experimental results showed that the MV pigment experiences a systematic color change with increasing temperatures, as expressed in the following chemical equation:

(1) $$\begin{array}{c} 2{\mathrm{MnNH}}_{4}P_{2}0_{7}\\ \left(\mathrm{dark}\;\mathrm{violet}\right) \end{array}\;\overset{\mathrm{RT}\;-\;370\;{}^{\circ}C}{↔}\;\begin{array}{c} {\mathrm{Mn}}_{2}P_{4}O_{13}{\left(\mathrm{NH}\right)}_{2}\\ \left(\mathrm{blue}\right) \end{array}\;\;\overset{410\;{}^{\circ}C}{\to }\;\;\begin{array}{c} {\mathrm{Mn}}_{2}P_{4}O_{12}\\ \left(\mathrm{grayish}\;\mathrm{yellow}\right) \end{array}$$

The dehydration process, which means the removal of water from pigment particles, occurred in between room temperature and 370 °C. Due to water driven out of the pigment particles, the color of the pigment changed from violet to reversible blue. When the pigment was exposed to the atmosphere again, it reacted with the ambient moisture to restore its original violet color. Immediately after 370 °C, the violet color started to diminish, and the white color appeared partially on the surface of the pigment particles. However, the color change was inadequate to be observed precisely until 400 °C. When the temperature reached 410 °C, the color of the pigment completely changed from violet to grayish yellow. The stable color was maintained even after heating to higher temperatures. This was an irreversible reaction associated with both the evaporation of water and the liberation of ammonia from the pigment particles at temperatures higher than 400 °C. That is, when cooled to room temperature, the MV pigment did not recover its original color.

**Figure 2:**
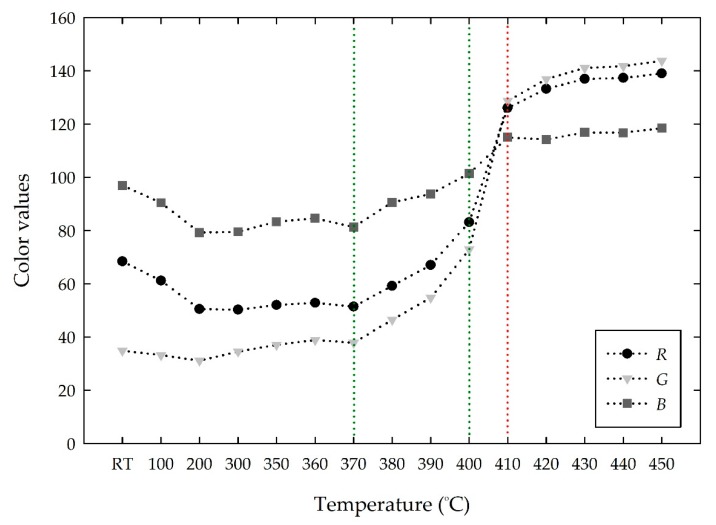
Changes in the *RGB* values of the MV pigment with increasing temperature.

In addition, the color change was evaluated using *RGB* and *Lab* color spaces. Figure 2 shows the change in the *RGB* values, averaged from the images at each temperature, with increasing temperature. The *RGB* values decreased slightly from (68, 34, 96) to (50, 31, 79) from room temperature to 200 °C, respectively, and the *RGB* values were almost constant with increasing temperature until 370 °C. After that, all the *RGB* values began to increase gradually toward the whiteness color intensity up to 400 °C. The *RGB* values showed a sudden hike to (126, 128, 114) at 410 °C, which is the transition point of a color change from violet to white. Above 410 °C, the RGB values were stable as an irreversible white color. As a result, the *RGB* values at 410 °C were approximately 1.85, 3.76, and 1.19 times those at room temperature, respectively.

**Figure 3:**
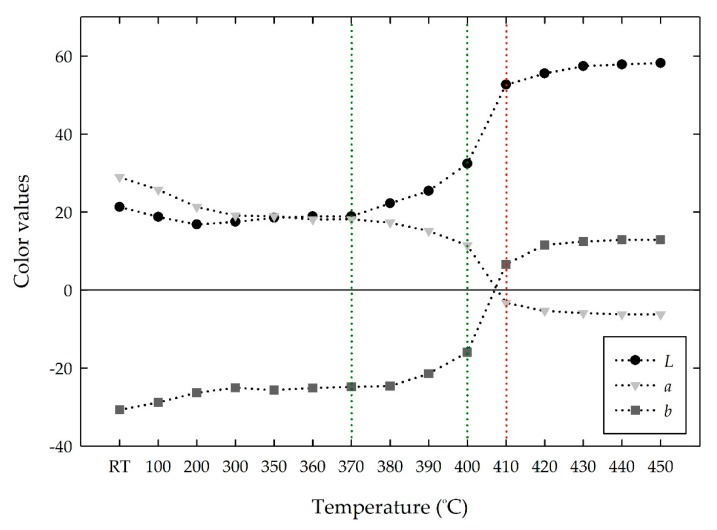
Changes in the *Lab* values of the MV pigment with increasing temperature.

Figure 3 shows the change in the color values in the *Lab* space. Similar to the *RGB* color values, the *Lab* values were relatively constant from room temperature to 370 °C. At higher temperatures, the *L* value began to gradually increase toward lightness; *b* value also gradually increased, while the *a* value decreased. At 410 °C, the *Lab* color values showed a rapid change to around (52, −3, 6), respectively. The color change means a change from darkness to lightness. That is, the lightness index of the *L* value increased by approximately 248% from 21 at room temperature. The *a* value decreased from positive (28 at room temperature) to negative (−3 at 410 °C), which indicates a greenness intensity, whereas the *b* value increased from negative (−30 at room temperature) to positive (6 at 410 °C), which indicates yellowness. These three *Lab* color values at 410 °C were combined to produce a grayish yellow color. Above 410 °C, little change in the *Lab* values was observed.

### 3.2. SEM Micrograph and EDX Analysis of the MV Pigment

**Figure 4:**
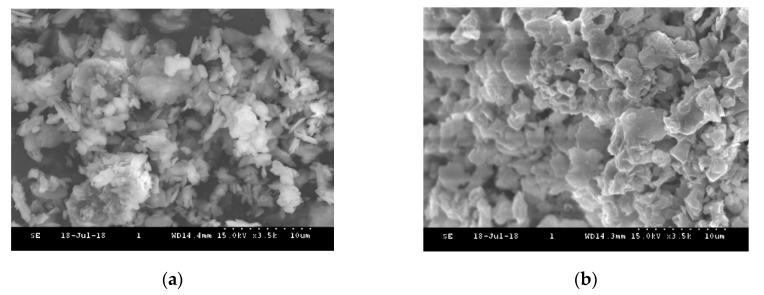
SEM images of the MV pigment: (**a**) Room temperature; (**b**) 410 °C.

For further characterization, SEM (S-4300 FE-SEM, Hitachi, Tokyo, Japan) was performed on the MV pigment at room temperature and at 410 °C to follow the microstructural changes because of the heat treatment. Figure 4 presents SEM images of the morphology and structure of the MV pigment. From the figure, the MV pigment at room temperature revealed a cluster of rod-shaped, spherical, and hexagonal crystal structures. As the temperature was increased to 410 °C, the rod-shaped and hexagonal crystal characteristics of the MV pigment tended to change through the loss of water and ammonia. The dehydration process at high temperatures disintegrated their edges and provided a cluster of ring-shaped void structures. 

**Figure 5:**
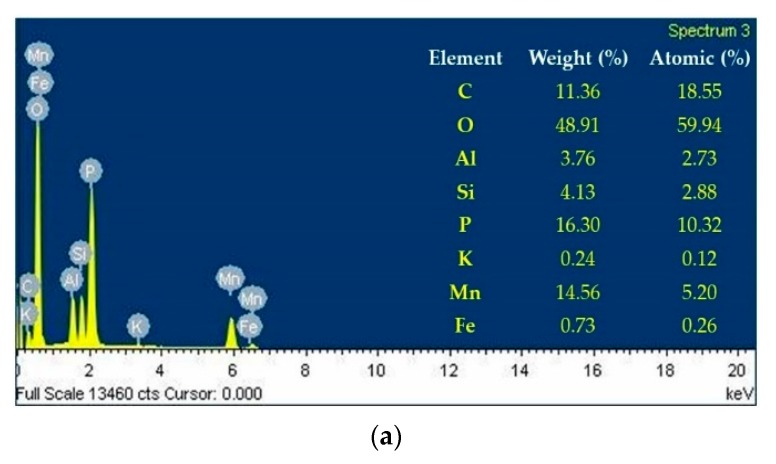

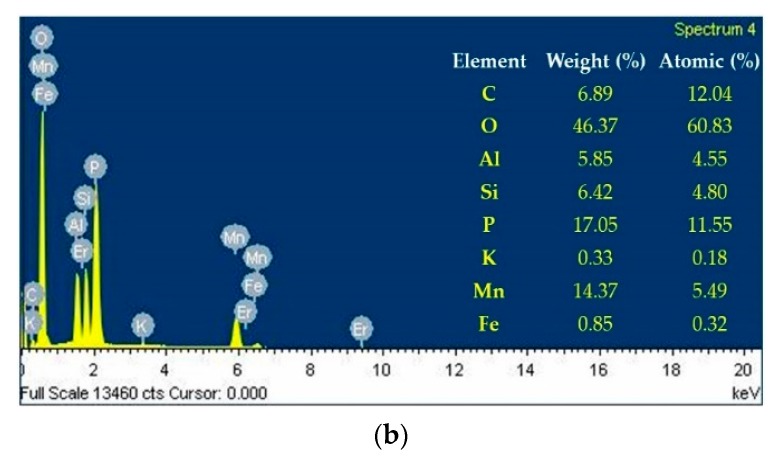
EDX analysis of the MV pigment: (**a**) Room temperature; (**b**) 410 °C.

EDX analysis (EMAX-7000, HORIBA EDX system, Kyoto, Japan) was carried out to assess the changes in the mineralogical composition of the samples caused by the increase in temperature, as displayed in Figure 5. In the unaltered sample at room temperature, the EDX pattern exhibited the presence of more intense peaks of C, O, P, and Mn with the mass contents of 11.36%, 48.91%, 16.30%, and 14.56%, respectively, and less intense Al, Si, K, and Fe peaks. When exposed to 410 °C, the mass of C, O, and Mn decreased by 39%, 5%, and 1.35%, respectively, and P increased by 4.6%, in which the mass contents of the C, O, P, and Mn changes to 6.89%, 46.37%, 17.05%, and 14.37%, respectively.

## 4. Application of MV Pigment with Cementitious Material

### 4.1. Thermochromic Change of White Cement Mixed Samples with MV Pigment

**Figure 6:**
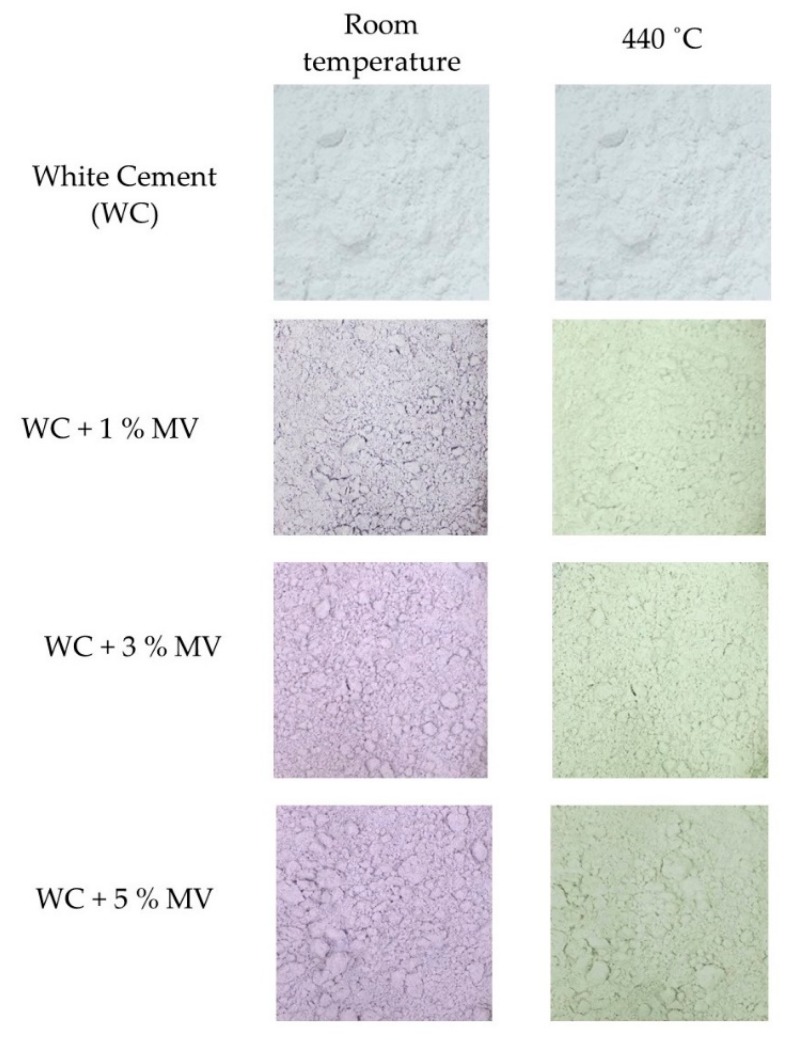
Color changes in the white cement and MV mixed samples exposed to different temperatures (Scale: 1000 × 1000 pixels).

White Portland cement is widely used in cement composites for the good exposure of color with pigments. Primarily, white cement remains a stable silver or pale white under high temperature conditions. Therefore, this study manufactured the white cement composite samples with the MV pigment. The MV pigment was separately added in 1%, 3%, and 5% of the weight of white cement. The mixed samples were heated individually in the furnace from room temperature to 450 °C. The photographs were recorded to examine the color of the samples at 100 °C, 200 °C, 300 °C, 350 °C, and 370 °C, and after that, every 10 °C up to 450 °C. Figure 6 shows the original and fully changed colors of the mixed samples at room temperature and 440 °C, respectively.

With the addition of white cement, the color of the pigment changed from violet to blue at room temperature. When heated, the mixed samples exhibited similar thermochromic changes as the pure MV pigment. Between room temperature to 410 °C, the mixed samples underwent a process of water removal from the pigment, but the color change was insufficient to be observed with the naked eye. Subsequently, the surface color of the mixed samples tended to fade away. When the temperature reached 440 °C, the mixed samples with 1%, 3%, and 5% of the MV pigment completely changed to a grayish green color. The color change was attributed to both the evaporation of water and the decomposition of ammonia from the pigment particles. The mixed samples did not recover their original color upon cooling to room temperature, indicating an irreversible color change. The color change occurred at 410 °C in the pure pigment but at 440 °C for the mixed samples. This might be due to the reduced temperature intrusion between the layers due to the mixing of white cement.

### 4.2. SEM Micrograph and EDX Analysis of White Cement Mixed Samples

**Figure 7:**
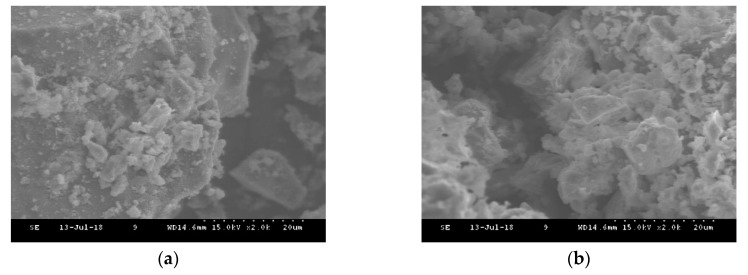
SEM images of the white cement: (**a**) Room temperature; (**b**) 440 °C.

**Figure 8:**
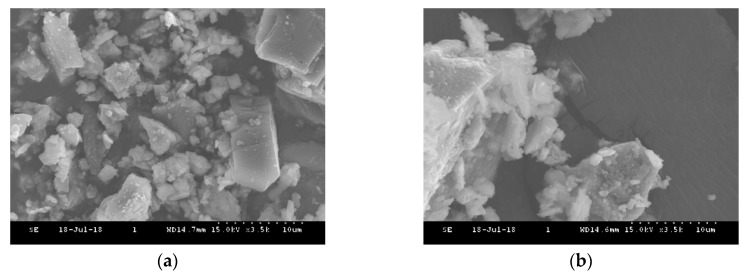
SEM images of the mixed samples: (**a**) Room temperature; (**b**) 440 °C.

Figure 7 shows SEM images of the white cement to characterize the changes in the morphology associated with high temperatures. At room temperature, the particles of the white cement have a structural diversity with globular shapes, including hexagonal, spherical, and some irregular tiny particles. When heated to 440 °C, a slight change was found due to the breakage of particles, and the white cement was relatively stable. Figure 8 presents SEM micrographs of the mixed sample at room temperature and at 440 °C, respectively. From the figure, the mixed sample at room temperature exhibits triangular, hexagonal, and spherical particles with a small cluster of rod-shaped structures due to the presence of MV pigment. At 440 °C, the hexagonal and rod-shaped cluster structures disappeared due to the dehydration of MV particles, which changed the mixed sample to irregular bundled structures.

Table 2 presents the results of EDX analysis for the white cement and the mixed samples. In the white cement, the samples are predominantly composed of C, O, and Ca, with the mass contents of 14.9%, 47.35%, and 25.09%, respectively. At 440 °C, the C, O, and Ca contents were 15.35%, 48.03%, and 26.85%, which correspond to an increase of approximately 3%, 1.5%, and 7%, respectively.

The mixed sample consists of high peaks of C, O, and Ca with the mass components of 17.18%, 40.43%, and 19.19%, respectively, and low peaks of Mg, Al, Si, P, S, Mn, Sn, Sb, I, and Fe. The mixed sample with MV pigment included new elements of P, Mn, Sn, Sb, and I, which consisted of 2.05%, 1.78%, 1.24%, 8.03%, and 2.21%, respectively. When the temperature reached 440 °C, the C increased by 41.4%, and the O and Ca contents decreased by 3.3 and 11%, respectively. The mass of the new elements of P, Mn, Sn, Sb, and I by the addition of MV at 440 °C changed to 1.54%, 1.72%, 0.89%, 6.67%, and 2.07%, which correspond to a 24%, 3%, 28%, 16%, and 6% decrease, respectively.

### 4.3. Thermochromic Analysis in the RGB Color Spaces

**Figure 9:**
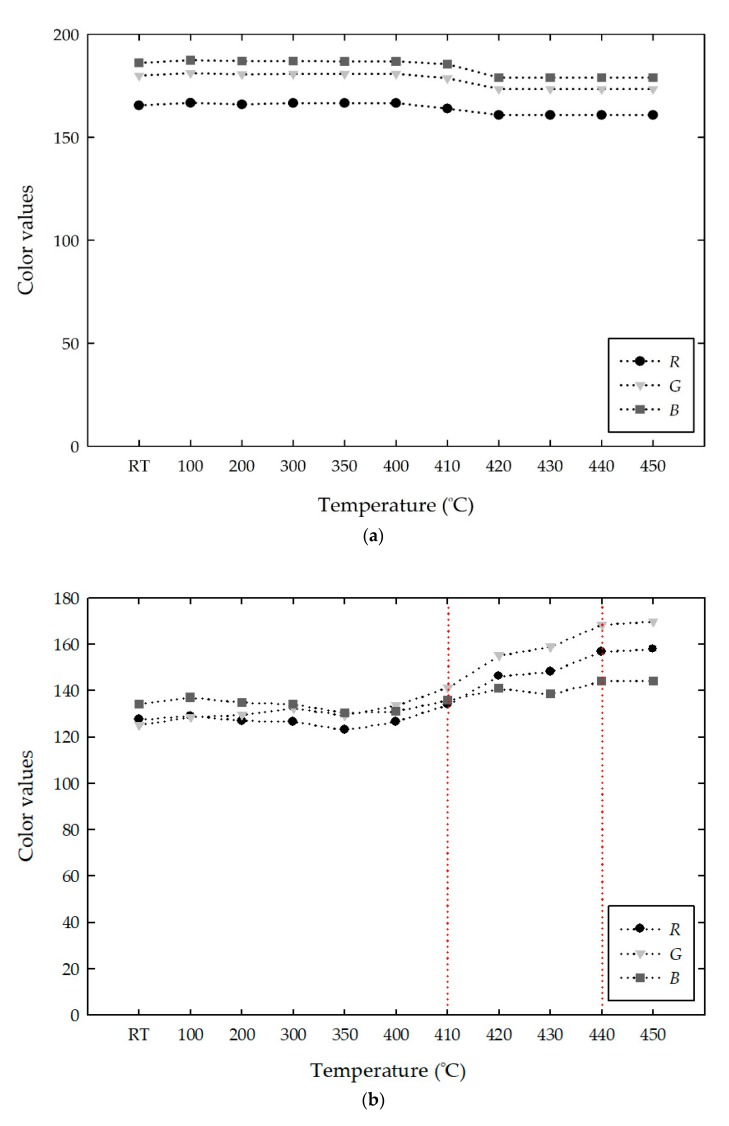

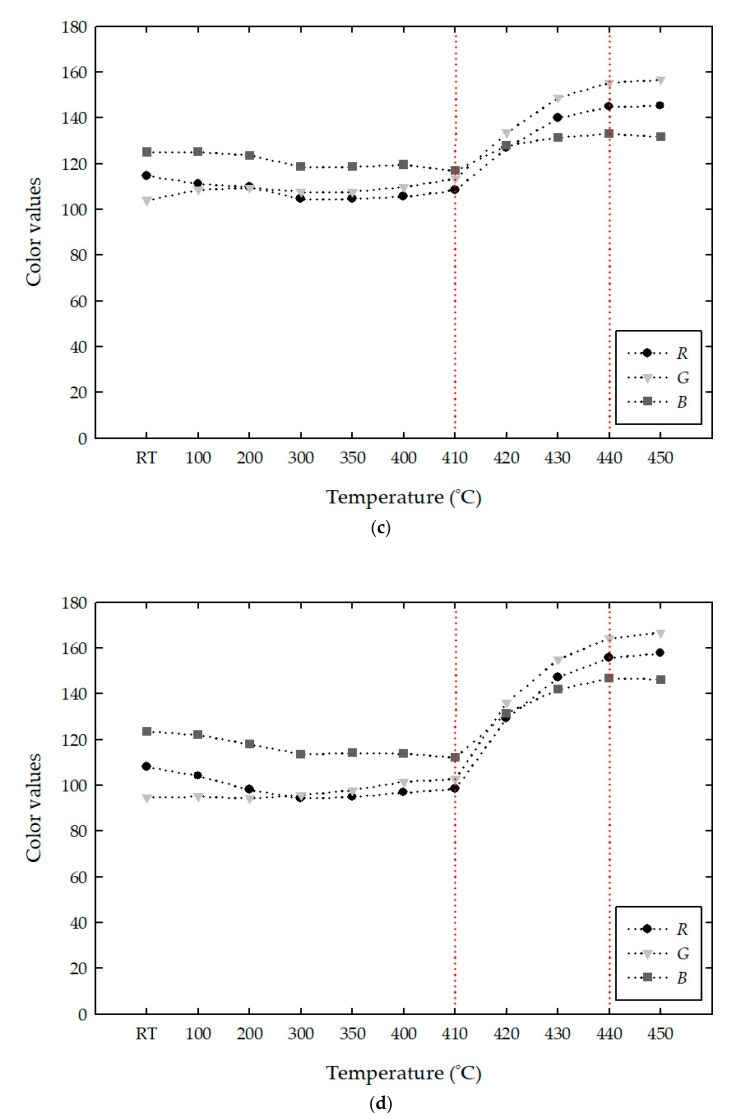
Changes in the *RGB* values of the white cement (WC) and MV mixed samples with increasing temperature: (**a**) WC; (**b**) WC + 1% MV; (**c**) WC + 3% MV; and (**d**) WC + 5% MV.

The color changes of the white cement mixed samples were evaluated using *RGB* and *Lab* color spaces. Figure 9 shows the changes in *RGB* values with increasing temperature. The white cement showed stable and high *RGB* values from room temperature to 450 °C, as shown in Figure 9a. At room temperature, the *RGB* color intensities of white cement were (165, 179, 186), which represents a grayish blue color. Heating of the white cement barely changed the color and thus displayed stable values with the mean *RGB* values of (164, 177, 183) from room temperature to 450 °C. Figure 9b–d show the changes in the *RGB* values of the mixed samples with increasing temperature. The addition of 1% pigment to white cement decreased the *RGB* values at room temperature from approximately (165, 179, 186) to (127, 125, 134), which corresponds to a 22% to 30% (R: 22%, G: 30%, B: 27%) decrease. In the mixed samples with 3 % and 5% pigments, the *RGB* values decreased by approximately 30% to 42% (*R*: 30%, *G*: 42%, *B*: 32%) and 33% to 47% (*R*: 34%, *G*: 47%, *B*: 33%), respectively. The decrease in the *RGB* values in the mixed samples indicates the disappearance of the whiteness intensity and the development of a mild violet color with the addition of 1%, 3%, and 5% pigments. With increasing temperature from room temperature to 410 °C, the mixed samples with 1%, 3%, and 5% pigments were almost stable with mean *RGB* values of approximately (127, 131, 133), (108, 108, 120), and (99, 97, 116), respectively, which are very close to those at room temperature. At higher temperatures, the *RGB* increased gradually toward the whiteness intensity, and the color completely changed at 440 °C. The *RGB* values of the mixed samples with 1%, 3%, and 5% MV at 440 °C were similarly (156, 168, 144), (145, 155, 133), and (157, 164, 146), respectively. Considering the reference *RGB* values of gray and green colors, the color obtained in the mixed samples at 440 °C can be defined as a grayish green color. Above 440 °C, the *RGB* values were stable and irreversible.

**Figure 10:**
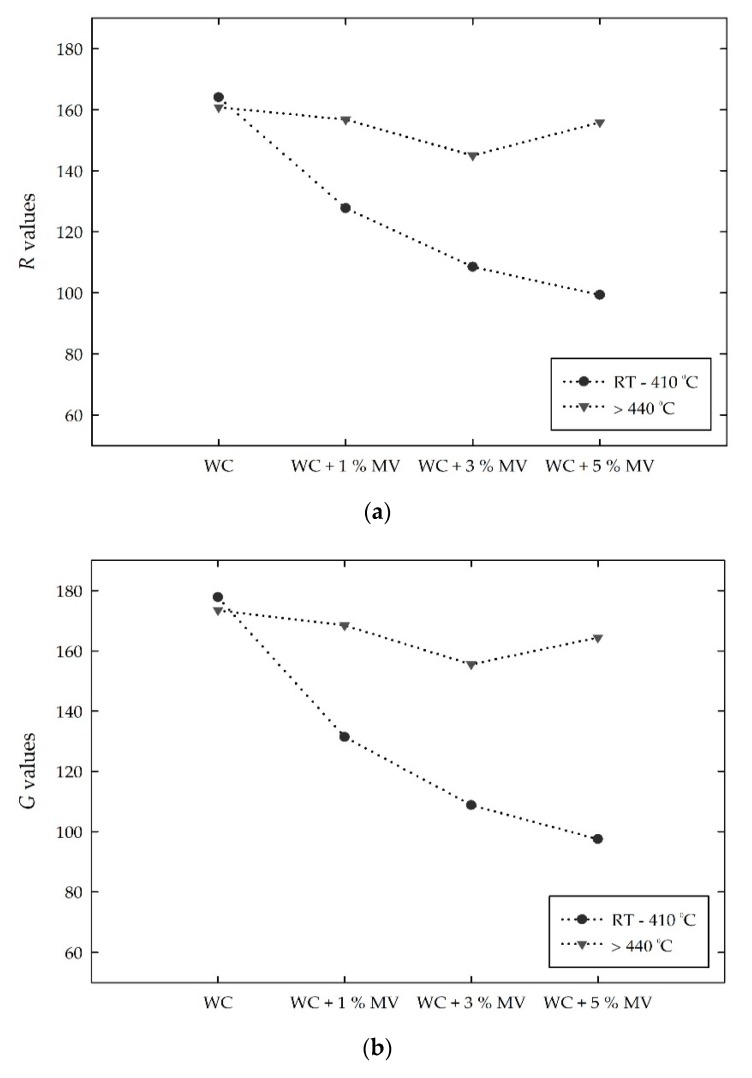

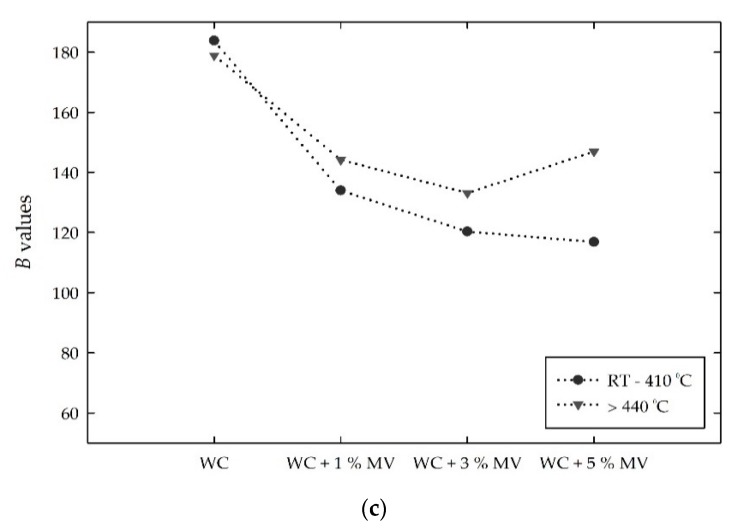
Comparisons of the *RGB* values for the white cement (WC) mixed samples with increasing temperatures: (**a**) *R* values; (**b**) *G* values; and (**c**) *B* values.

With increasing pigment content, the mean *RGB* values of the mixed samples from room temperature to 410 °C, in which the color of the samples was stable, were compared with those at a critical temperature of 440 °C, as shown in Figure 10. Compared to *R* values in between room temperature and 410 °C, those at 440 °C increased by approximately 22%, 33%, and 55% in the WC + 1% MV, WC + 3% MV, and WC +5 % MV specimens, respectively, as shown in Figure 10a. At a critical temperature of 440 °C, which completely changed to a grayish green color, the mixed samples with 1%, 3%, and 5% pigment showed *R* values in the range of 144 to 155. This indicates that the *R* value decreased due to the inclusion of pigment at room temperature, but increased to a similar range at a critical temperature of 440 °C. Figure 10b shows the change in *G* values for the white cement mixed samples. With increasing MV pigment, the *G* values decreased at room temperature. At 440 °C, the *G* values of the white cement with 1%, 3%, and 5% of MV pigment increased by 28%, 42%, and 68%, respectively, and showed a similar range of 155 to 168, irrespective of the content of MV pigment. Figure 10c shows the change in the *B* value for the white cement mixed samples. The *B* intensities, which were also stable between room temperature and 410 °C, decreased from approximately 183 to 116 with increasing content of MV pigment from zero to 5%. The *B* value at the critical temperature of 440 °C increased to 144, 133, and 146, which corresponds to a 7%, 10%, and 25% increase for the white cement samples with 1%, 3%, and 5%, respectively. The addition of pigments decreased the *B* values, but the amount of the increase is relatively small compared to that of the *R* and *G* values.

**Figure 11:**
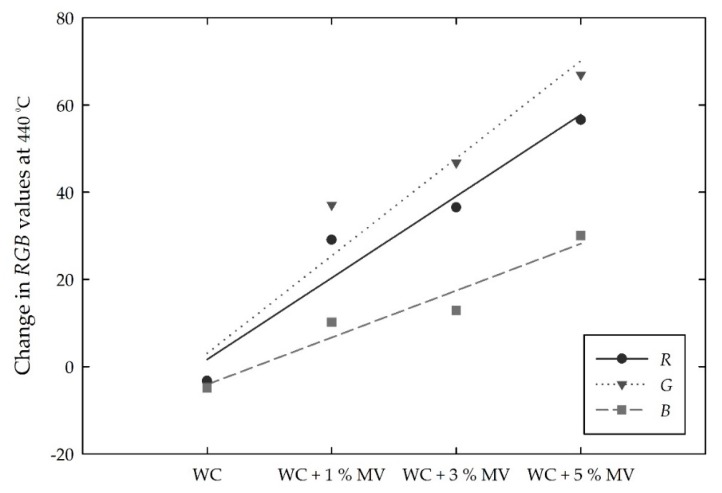
Changes in the *RGB* values for the white cement (WC) mixed samples with increasing MV pigment contents at 440 °C.

In the *RGB* color intensities, the incorporation of MV pigment particles in 1%, 3%, and 5% decreased the *RGB* intensities, which were stable between room temperature and 410 °C. The color intensities began to increase from 410 °C and completely changed to grayish green at 440 °C, at which point little difference of the *RGB* values was found among the white cement mixed samples. That is, with increasing pigment content, the *RGB* intensities decreased at room temperature but obtained relatively constant values at 440 °C. The *RGB* values and color intensities after 440 °C exhibited a slight difference, irrespective of the amount of MV pigment added to the white cement. Therefore, the magnitude of the change in the *RGB* values at 440 °C is also discussed. The changing magnitude of the *RGB* values mainly depends on the pigment to white cement ratio, as shown in Figure 11. With increasing addition of MV pigment from 1% to 5%, the magnitude of the change in the *RGB* almost linearly increased. According to the linear trend analysis, the *R* and *G* values showed a 10.9 and 13.0 increase at 440 °C per 1% addition of MV pigment to white cement, respectively. The approximately 6.5 increase in the *B* value per 1% MV pigment was relatively small. This suggests that the *R* and *G* values, which show a higher change at 440 °C than the *B* value, would be an index to instantly identify and evaluate cementitious materials subjected to a critical high temperature.

**Figure 12:**
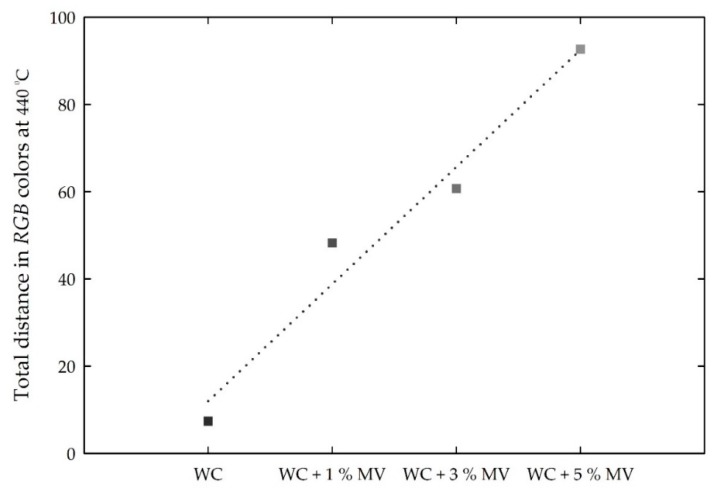
Total distance in the *RGB* intensities for the white cement (WC) mixed samples with increasing MV pigment content at 440 °C.

The color changes of the white cement mixed samples are related to the increasing number of *RGB* values. Therefore, the total color changes in the *RGB* color intensities are defined as the Euclidean distance $\left(\bar{d}\right)$ using the following equation:

(2) $$\bar{d}\;=\;\sqrt{{\left(\Delta R\right)}^{2}+{\left(\Delta G\right)}^{2}+{\left(\Delta B\right)}^{2}}$$

where Δ*R*, Δ*G*, and Δ*B* represent the differences of the *R*, *G*, and *B* values, respectively, 440 °C and those between room temperature to 410 °C. These also show a linear increase with increasing MV pigment ratio, as shown in Figure 12. The increasing rate of $\bar{d}$ is approximately 14.7 per 1% addition of MV pigment to white cement. As a result, the total color changes of white cement mixed with MV pigment can be effectively determined using the increases in *RGB* color values and $\bar{d}$.

### 4.4. Thermochromic Analysis in the Lab Color Spaces

**Figure 13:**
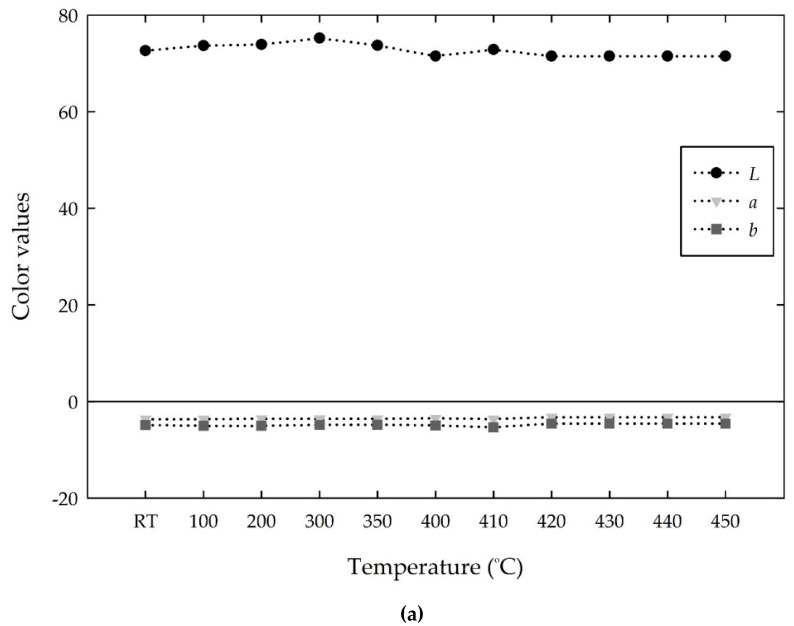

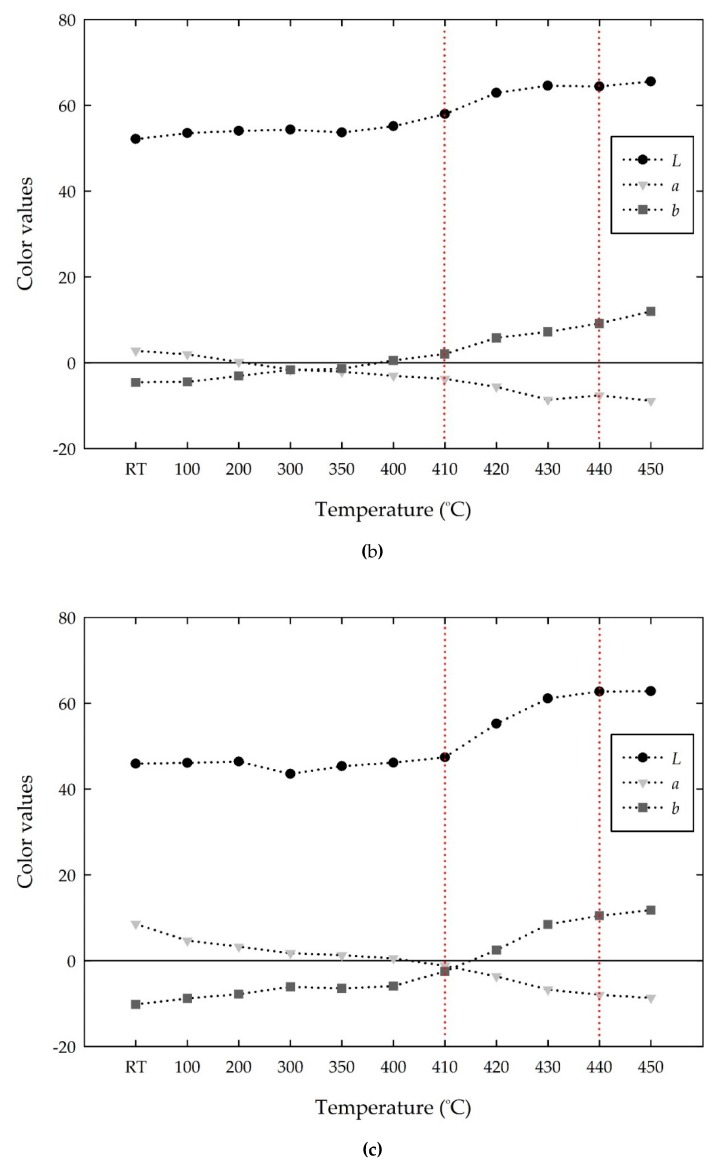

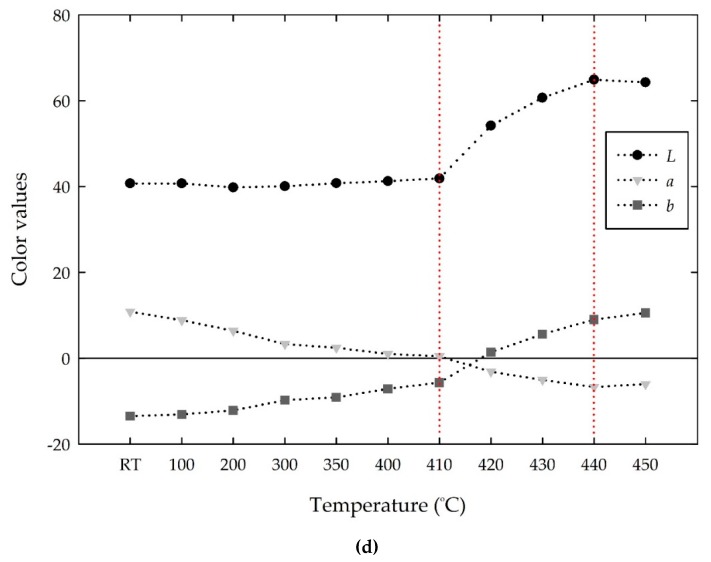
Changes in the *Lab* values of the white cement (WC) and MV mixed samples with increasing temperature: (**a**) WC; (**b**) WC + 1% MV; (**c**) WC + 3% MV; and (**d**) WC + 5% MV.

Figure 13 shows the changes in the *Lab* values of the white cement mixed samples with increasing temperature. The white cement exhibited a constant high *L* value of approximately 72, and the *a* and *b* values present negative constant values of approximately −3 and −4, respectively, with increasing temperature from room temperature to 450 °C, as shown in Figure 13a. This suggests that the white cement is stationary with increasing temperature. The addition of MV pigment decreased the *L* and *b* values, whereas it increased the *a* value at room temperature, as shown in Figure 13b–d. The mixed samples with 1%, 3%, and 5% of the MV pigment showed a decrease in *L* value from 72 to 52, 45, and 40, corresponding to a 28%, 36%, and 43% decrease, respectively. The *a* value, which was −3 at room temperature for white cement, changed to positive values (redness degrees) of 2, 8, and 10 for the mixed samples with 1%, 3%, and 5% of MV pigment, respectively. In contrast, the *b* intensity, which was negative for the white cement, showed a further decrease to a negative value (blueness degree). The change in the *Lab* values influences the development of color on the mixed samples. That is, the WC + 1% MV sample with the Lab of (52, 2, −4) represents a dark grayish blue color, and WC + 3% MV and WC + 5% MV samples with the *Lab* of (45, 8, −10) and (40, 10, −13), respectively, illustrate dark grayish violet color at room temperature. As the temperature increased from room temperature to 410 °C, the *L* value was relatively constant, the *a* value gradually decreased from positive to negative, and the b value increased from negative to positive. However, the changes in the *Lab* values were too low to observe the color change of the mixed samples until 410 °C. 

After 410 °C, the color of the samples started to show a grayish green color with increasing *L* value and changing *a* from positive to negative and *b* from negative to positive values. The increase in *L* value increases the lightness intensity, and the decrease in *a* and increase in *b* indicate the greenness and yellowness intensities, respectively. At 440 °C, the mixed samples completely changed to a grayish green color and showed Lab values of (64, −7, 9) for 1% MV, (62, −7, 10) for 3% MV, and (64, −6, 9) for 5% MV samples. As discussed in the *RGB* color spaces, the *Lab* values were also similar at the critical temperature of 440 °C, irrespective of the amount of MV pigment added to the white cement. Above the critical temperature, the mixed samples exhibited stable and invariable *Lab* values.

**Figure 14:**
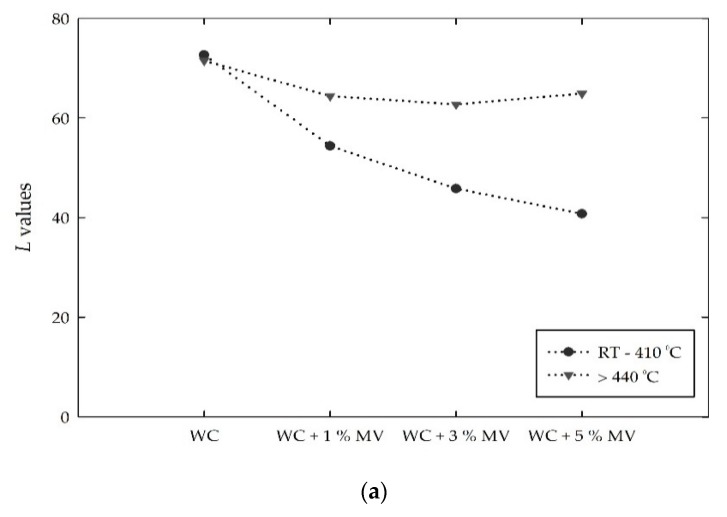

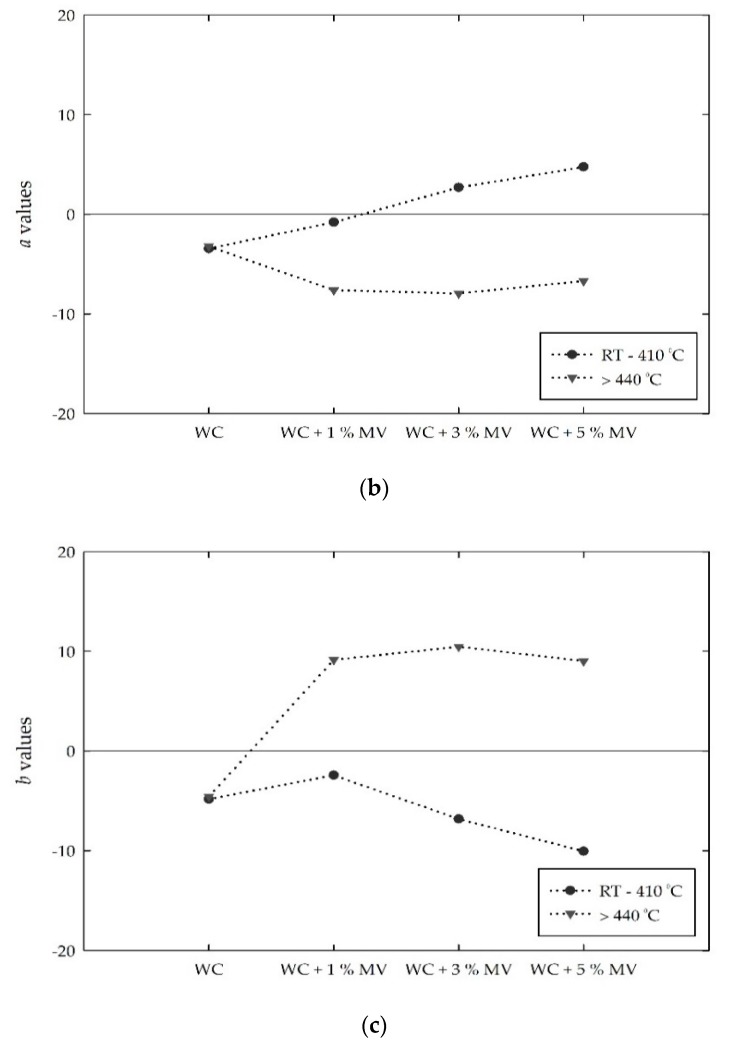
Comparisons of the *Lab* values for the white cement (WC) mixed samples with increasing temperature: (**a**) *L* values; (**b**) *a* values; and (**c**) *b* values.

Figure 14 compares the *Lab* values of the mixed samples averaged from room temperature to 410 °C with those at a critical temperature of 440 °C. As shown in Figure 14a, the *L* value (or, the lightness intensity) decreased when the pigment concentration was increased from zero to 5%. However, the *L* value at 440 °C showed a similar level, ranging from 62 to 64, which indicates a grayish color. Figure 14b shows the change in *a* value. Compared to those between room temperature and 410 °C, the *a* values at the critical temperature of 440 °C decreased from positive to negative, resulting in a very similar range of −7 to −6 for all three mixed samples. In contrast, the *b* value increased from negative to positive, ranging from 9 to 10, which illustrates the increase in yellowness intensity, when the mixed samples reached the critical temperature of 440 °C, as shown in Figure 14c.

**Figure 15:**
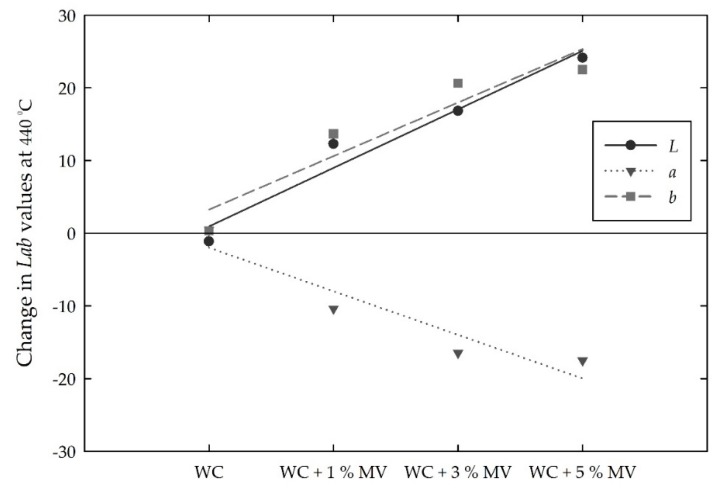
Changes in the *Lab* values for the white cement (WC) mixed samples with increasing MV pigment content at 440 °C.

Based on the mean *Lab* values between room temperature and 410 °C, in which no major change was obtained in all the white cement mixed samples, Figure 15 shows the magnitude of the change in the *Lab* values at 440 °C. The *L* and *b* values changed to positive values, and the *a* values changed to negative values. That is, with increasing MV pigment from zero to 5%, the *L* and *b* values increased almost linearly from −1.9 to 23.8 and from 0.3 to 20.6, respectively, whereas the *a* value decreased from 0.4 to −11.1. A linear trend analysis showed that the magnitude of the *L* and *b* values at 440 °C increased by approximately 4.7 and 3.6, respectively, and the *a* value, the change of which was relatively small, decreased by approximately 2.1 per 1% increase in MV pigment. 

**Figure 16:**
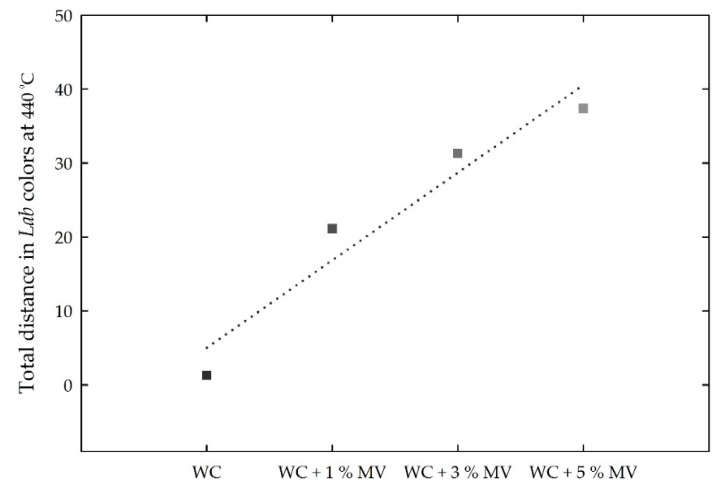
Total distance in the *Lab* intensities for the white cement (WC) mixed samples with increasing MV pigment content at 440 °C

In addition, the total changes in the *Lab* intensities at the critical temperature of 440 °C were calculated, as shown in Figure 16. Similar to Equation (2), the total change was defined as the Euclidean distance ($\bar{d}$), using the difference between the mean *Lab* values from room temperature to 410 °C and those at 440 °C. As the content of the MV pigment added to white cement increased from zero to 5%, $\bar{d}$ tended to increase almost linearly at a rate of approximately 5.8 per 1% addition of MV pigment to white cement. Along with *RGB* intensities, the *Lab* changes can be used to determine the color change at a critical temperature with the white cement-mixed samples containing MV pigment.

## 5. Conclusions

This study proposed an irreversible thermochromic cementitious material using MV pigment. According to the thermochromic tests, the MV pigment underwent a reversible change from violet to blue at temperatures lower than 400 °C, but the color change was barely discernable by the naked eye. When the temperature reached 410 °C, the MV pigment completely changed from violet to grayish yellow, which is associated with the evaporation of water and the liberation of ammonia from the pigment particles. The changed color was maintained at temperatures higher than 410 °C and when the temperature was returned to room temperature.

In the analysis of MV pigment in the *RGB* and *Lab* color spaces, the values were relatively constant from room temperature to 370 °C. At higher temperatures, the *RGB* values increased gradually toward the whiteness color intensity until 400 °C. The *L* and *b* values also gradually increased after 370 °C, while the *a* value decreased. At 410 °C, when the color completely changed from dark violet to grayish yellow, the *RGB* and *Lab* provided a sudden change and remained constant after the critical temperature of 410 °C. In particular, the *a* value changed from positive to negative, and the *b* value changed from negative to positive.

The MV pigment was then mixed with white cement at ratios of 1%, 3%, and 5% of the mass of the white cement. The color of the mixed samples was dark grayish blue and violet, depending on the content of MV pigment. The colors at room temperature were retained until 410 °C. Hence, the *RGB* and *Lab* intensities were relatively stable and constant. The color started to change at temperatures higher than 410 °C and completely turned to grayish green at 440 °C. In accordance with the change in color, the *RGB* increased toward the whiteness intensity. Compare to those before the thermochromic change occurred, the *RGB* values at 440 °C increased by approximately 22%–55%, 28%–68%, and 7%–25%, respectively. The *RGB* values almost linearly increased with the increasing content of MV pigment.

Similarly, the *L* value increased by approximately 23%–60% at 440 °C. The *a* value changed from positive to negative, and the *b* value changed from negative to positive. The changes in the Lab values also increased almost linearly with increasing MV pigment content. According to the linear trend analyses, the magnitude of the *Lab* values at 440 °C changed by approximately 4.7, 3.6, and −2.1, respectively, and the *RGB* values increased by approximately 10.9, 13.0, and 6.5, respectively, per 1% increase in MV pigment. In addition, the total changes in the *RGB* and *Lab* color intensities at the critical temperature of 440 °C, defined as the Euclidean distance, showed the increasing rates of approximately 14.7 and 5.8 per 1% addition of MV pigment to white cement.

However, the level of the *RGB* and *Lab* values and the color intensity of the mixed samples at 440 °C were in very similar ranges, irrespective of the amount of MV pigment added to white cement. Above a critical temperature of 440 °C and back to room temperature, the mixed samples exhibited stable and irreversible *RGB* and *Lab* values. Therefore, with a direct visual inspection of the distinct color change, the changes in the *RGB* and *Lab* values can provide an instant and promising index to identify the magnitude and dispersion of critical temperatures in cementitious materials and structures with MV pigment.

SEM images showed that the rod-shaped and hexagonal crystal structures of the MV pigment at room temperature were modified to a cluster of void structures at 410 °C. The EDX analysis results of the MV pigment, comprised of 11.36%, 48.91%, 16.30%, and 14.56% of C, O, P and Mn, respectively, changed to 6.89%, 46.37%, 17.05%, and 14.37%, after exposure to 410 °C, respectively. In the SEM images, the white cement, composed of hexagonal and spherical shapes with some irregular tiny particles, was relatively stable with slight changes due to the breakage of particles at 440 °C. The mixed samples consisted of triangular, hexagonal, and spherical particles with a small cluster of rod-shaped structures at room temperature. At 440 °C, the hexagonal and rod-shaped cluster structures disappeared due to the dehydration of MV particles, which changed into irregular bundled structures. In the EDX analysis, the white cement, which is mainly composed of C, O, and Ca with the mass contents of 14.9%, 47.35%, and 25.09% at room temperature, increased by 3%, 1.5%, and 7%, respectively, at 440 °C. The mixed sample exhibited C, O, and Ca with the mass components of 17.18%, 40.43%, and 19.19%, respectively, and P, Mn, Sn, Sb, and I with the mass of 2.05%, 1.78%, 1.24%, 8.03%, and 2.21%, respectively, due to the addition of MV pigment. At 440 °C, the mass of C, O, and Ca changed to 24.29%, 39.09%, and 17.05%, respectively, and that of P, Mn, Sn, Sb, and I decreased by 24%, 3%, 28%, 16%, and 6%, respectively.

## Figures and Tables

**Table 1:** Typical characteristics of MV pigment.

Parameter	Characteristics
Color	Violet
Chemical characterization	Manganese III ammonium pyrophosphate
Density	2.7 ~ 2.9 kg/m^3^
Bulk density	0.60 g/cm^3^
Average particle size	2.30 µm
pH value	2.5 ~ 4.7
Thermal decomposition	>400 °C

**Table 2:** EDX analysis of the white cement mixed samples—weight (%).

Element	White Cement (%)		WC + MV Sample (%)	
	Room Temperature	440 °C	Room Temperature	440 °C
**C**	14.90	15.35	17.18	24.29
**O**	47.35	48.03	40.43	39.09
**Mg**	2.15	2.50	2.53	3.98
**Al**	1.19	0.65	1.47	0.54
**Si**	4.22	2.52	2.84	1.09
**P**	-	-	2.05	1.54
**S**	1.06	0.99	1.06	0.51
**K**	0.39	-	-	-
**Ca**	25.09	26.85	19.19	17.05
**Mn**	-	-	1.78	1.72
**Sn**	-	-	1.24	0.89
**Sb**	-	-	8.03	6.67
**I**	-	-	2.21	2.07
**Ti**	-	0.25	-	-
**Fe**	2.07	2.88	-	0.57
**Ge**	0.47	-	-	-
**Zr**	0.91	-	-	-
